# The rise and fall of CB_1_ receptor antagonists: possible future perspectives

**DOI:** 10.1186/1471-2210-11-S2-A55

**Published:** 2011-09-05

**Authors:** György Bagdy

**Affiliations:** 1 Department of Pharmacodynamics, Semmelweis University, 1089 Budapest, Hungary

## Abstract

Cannabinoid type-1 (CB_1_) receptor antagonists were among the most promising drug targets in the last decade. They have been explored and found to be effective as therapeutic agents for obesity and related cardiometabolic problems, including e.g. dyslipidaemias, diabetes, and metabolic syndrome. However, the use of rimonabant, the first marketed CB_1_ receptor antagonist, has been suspended due to its anxiogenic and depressogenic side effects, which were present in about 20–30% of the patients, i.e. a 2.5–3-fold increase compared to placebo. Since some other anti-obesity drugs like dexfenfluramine or sibutramine were also suspended, the unmet need for drugs that reduce body weight became enormous. One approach that emerged was the development of peripheral CB_1_ receptor antagonists that poorly cross the blood brain barrier, the second, the development of neutral antagonists instead of inverse agonists, and the third, the selection of the patient population with reduced risk for psychiatric side effects. An analysis regarding peripheral and central mechanisms involved in the effects of CB_1_ receptor antagonists strongly suggest that central mechanisms are more or less involved in most cardiometabolic therapeutic effects and thus, among patients with unsatisfactory therapeutic response to compounds with peripheral action, centrally acting antagonists may be needed. Based on our existing knowledge concerning the role of genetic, phenotypic and environmental factors the selection of persons who are at no or low risk for psychiatric adverse effects may be possible. Molecular mechanisms and receptors involved in the effects of stress and anxiety-related neurocircuitries sensitive to CB_1_ receptor antagonists, like the serotonergic and noradrenergic systems which regulate the synthesis of the endocannabinoid 2-arachydonoyl-glycerol mediated by 5-HT_2C_ and α_1_ receptors can be identified [1]. Furthermore, variants of the serotonin transporter and the CB_1_ receptor genes have been shown to modulate stress-induced anxiety in human studies [1]. In conclusion, development of peripherally acting, or the use of personalised medicine for centrally acting CB_1_ receptor antagonists are promising approaches with diverse advantages.
